# Cardiogel: A Nano-Matrix Scaffold with Potential Application in Cardiac Regeneration Using Mesenchymal Stem Cells

**DOI:** 10.1371/journal.pone.0114697

**Published:** 2014-12-18

**Authors:** Rajalakshmi Santhakumar, Prasanna Vidyasekar, Rama Shanker Verma

**Affiliations:** Stem cell and Molecular Biology Lab, Department of Biotechnology, Indian Institute of Technology Madras (IITM), Chennai, Tamil Nadu, India; The University of Adelaide, Australia

## Abstract

3-Dimensional conditions for the culture of Bone Marrow-derived Stromal/Stem Cells (BMSCs) can be generated with scaffolds of biological origin. Cardiogel, a cardiac fibroblast-derived Extracellular Matrix (ECM) has been previously shown to promote cardiomyogenic differentiation of BMSCs and provide protection against oxidative stress. To determine the matrix composition and identify significant proteins in cardiogel, we investigated the differences in the composition of this nanomatrix and a BMSC-derived ECM scaffold, termed as ‘mesogel’. An optimized protocol was developed that resulted in efficient decellularization while providing the maximum yield of ECM. The proteins were sequentially solubilized using acetic acid, Sodium Dodecyl Sulfate (SDS) and Dithiothreitol (DTT). These proteins were then analyzed using surfactant-assisted in-solution digestion followed by nano-liquid chromatography and tandem mass spectrometry (nLC-MS/MS). The results of these analyses revealed significant differences in their respective compositions and 17 significant ECM/matricellular proteins were differentially identified between cardiogel and mesogel. We observed that cardiogel also promoted cell proliferation, adhesion and migration while enhancing cardiomyogenic differentiation and angiogenesis. In conclusion, we developed a reproducible method for efficient extraction and solubilization of *in vitro* cultured cell-derived extracellular matrix. We report several important proteins differentially identified between cardiogel and mesogel, which can explain the biological properties of cardiogel. We also demonstrated the cardiomyogenic differentiation and angiogenic potential of cardiogel even in the absence of any external growth factors. The transplantation of Bone Marrow derived Stromal/Stem Cells (BMSCs) cultured on such a nanomatrix has potential applications in regenerative therapy for Myocardial Infarction (MI).

## Introduction

Myocardial Infarction (MI) accounts for 50% of all Cardiovascular Heart Disease (CVHD)-related mortality and morbidity in the developing world [1], [2]. MI results in substantial loss of cardiomyocytes, causing irreparable damage to the myocardium [3]. Following MI, normal healing response is initiated during which the damaged myocardium is replaced by fibrotic scar tissue. This, however, leads to poor ventricular activity (reduced ejection fraction), ultimately causing heart failure and death [3]–[5]. Adult cardiomyocytes are terminally differentiated and do not replicate after injury, which results in irreversible loss of cardiac function in the infarcted region [6]. Recently, stem cell-based therapy has emerged as a promising approach to restore the original cardiac function in the infarcted/damaged myocardium [7]–[9]. Adult stem cell-mediated cardiac repair follows two strategies: Transplantation of adult stem cell-derived cardiomyocytes differentiated *in vitro* or transplantation of non-committed stem cells along with biochemical cues for *in situ* differentiation. These transplanted cells eventually integrate with the host tissue restoring functional myocardium [10]. The differentiation and host integration of the transplanted stem cells can be promoted using specialized three-dimensional scaffolds that provide support and biochemical stimuli for cells to attach, differentiate and organize into tissues [4], [11].

Cardiogel is a natural, heterogeneous Extra Cellular Matrix (ECM) scaffold derived from *in vitro* cultured cardiac fibroblasts. Cardiogel has been known to improve cardiomyocyte growth and maturation. Bone Marrow derived Stromal/Stem Cells (BMSCs) cultured on their own secreted ECM do not demonstrate protection against oxidative stress or cardiomyogenic differentiation; but BMSCs cultured on cardiogel showed increased cell proliferation and adhesion, enhanced cardiomyogenic differentiation and protection against oxidative stress [12]–[17]. However, the ECM components that contribute to the biological properties of cardiogel have not yet been completely characterized. These ECM components can be identified using comparative proteomic analysis of cardiogel in comparison with mesogel, a BMSC-derived ECM scaffold. However, such proteomic analyses require a substantial amount of completely solubilized matrix protein without containing any interfering substances such as detergents and intracellular contaminations. Therefore, our aim was to develop a suitable protocol for isolation, extraction and solubilization of the decellularized matrix, which will be compatible with proteomic analysis.

Comparative proteomic analysis using nano-liquid chromatography tandem-mass spectrometry (nLC-MS/MS) analysis with mesogel as control was used to identify unique ECM components of cardiogel, which may explain cardiogel's biological properties such as heightened protection against oxidative stress and enhanced cardiomyogenic differentiation [16], [17]. Furthermore, biological properties of cardiogel such as the cytocompatibility, potential for cardiomyogenic differentiation and angiogenesis were evaluated to validate cardiogel as a potential scaffold for cardiac regeneration.

## Materials and Methods

### Isolation and culture of cardiac fibroblasts, BMSCs and EA.hy926 cells

Cardiac fibroblasts were isolated from 1–3 days old Neonatal Swiss albino mice by explant culture technique followed by differential trypsinization and BMSCs were isolated from 6–8 week old Swiss albino mice (∼20 g) by modification of Soleimani & Nadri protocol [18]. All procedures, approved by the Institutional Animal Ethics Committee (IIT Madras, India) and the Committee for the Purpose of Control and Supervision of Experiments on Animals, Government of India, were performed in accordance with the Rule 5(a) of the Breeding and Experiments on Animals (Control and Supervision; 1998). EA.hy926 cells (ATCC CRL-2922) were a kind gift from Dr. Madhulika Dixit, Vascular Biology Lab, Department of Biotechnology, Indian Institute of Technology Madras (IITM), India. The cells were incubated at 37°C in a humidified atmosphere containing 5% CO_2_ with medium containing DMEM/F12 (1∶1) medium supplemented with FBS (20% for cardiac fibroblasts; 10% for BMSCs and EA.hy926 cells), 4.00 mM L-glutamine, 1000 mg/l glucose, 110 mg/l sodium pyruvate, 100 U/ml penicillin, 100 mg/ml streptomycin and 0.25 mg/ml amphotericin (Life Technologies, USA). The isolated cardiac fibroblasts and BMSCs were characterized using Immunocytochemistry (ICC).

### Optimization of ECM Isolation

The tissue culture plates were incubated with 0.25% gelatin solution (2 ml/20 cm^2^) at 37°C overnight, following which, the gelatin solution was removed, leaving behind a film of gelatin over the surface. The plates were exposed to ultraviolet (UV) rays of ∼62 µW/cm^2^ intensity for 90 min to enhance cross-linking. Cardiac fibroblasts (PN 3 to 12) were seeded on 0.25% gelatin pre-coated plates at a density of ∼1×10^4^ cells per cm^2^ and cultured in the same medium with 10 µg/ml insulin. Decellularization was carried out after the cells reach 95%–100% confluency (3–4 days) using four different protocols. The solutions and buffers used for decellularization were prepared fresh, filter sterilized and pre-warmed to 37°C before use.

#### Protocol I

The cells were washed with DPBS and incubated with 1 ml of Extraction Buffer (20 mM Ammonium Hydroxide (NH_4_OH) and 0.5% Triton X 100 (TX100)) at RT for 20–50 s with consistent, gentle shaking to dislodge only cells without detaching the ECM followed by three washes with DPBS.

#### Protocol II

The cells were washed with DPBS and incubated with 1 ml of Extraction Buffer (50 mM NH_4_OH and 0.05% TX100) at RT for 15–30 s with consistent, gentle shaking to dislodge only cells without detaching the extracellular matrix, followed by single wash with 50 mM NH_4_OH and three washes with DPBS.

#### Protocol III

The cells were washed with DPBS and incubated with 0.5 mM EDTA (2 ml/20 cm^2^) at 37°C for 120 min. Following incubation, the EDTA solution along with the dislodged cells was removed and the plates were washed with fresh EDTA solution thrice and DPBS thrice.

#### Protocol IV

The cells were washed with DPBS and incubated with 2 mM EDTA in DPBS (2 ml/20 cm^2^) at 37°C for 90 min. The EDTA solution along with dislodged cells was removed and the plates were washed with fresh EDTA solution thrice. 1 ml of Extraction Buffer (50 mM NH_4_OHand 0.05% TX100) was added and incubated at RT for 10–30 s with consistent, gentle shaking to dislodge only cells without detaching the extracellular matrix, followed by single wash with 50 mM NH_4_OH and three washes with DPBS.

Following decellularization, the cardiogel coated plates were stored at 37°C in DPBS. The procedures were repeated with cardiac fibroblasts seeded on gelatin non-coated plates.

### ECM Extraction for Proteomic Studies

The cardiogel obtained through the 4 protocols were subjected to the treatment of 5% Acetic acid at 4°C overnight. The acetic acid was then replaced with a buffer containing 125 mM Tris (pH 6.8), 0.1% Sodium Dodecyl Sulphate (SDS), 5% glycerol. 1% Dithiothreitol (DTT), Protease Inhibitor Cocktail (PIC), Phenyl Methane Sulfonyl Fluoride (PMSF) and incubated at 37°C for 60 min following which the ECM was scraped off using cell scraper. This procedure was repeated thrice. Both the acetic acid fractions and the buffer fractions were combined and precipitated by incubation with five times volume of pre-chilled acetone at −20°C overnight. The precipitated protein fraction is collected by centrifugation at 7000 g, 4°C for 15 min. The pellet was air dried and dissolved in 0.1% RapiGest SF surfactant (Waters, Manchester, UK) in 50 mM ammonium bicarbonate (pH 7.4). Mesogel was isolated from BMSCs (PN 3 to 12) seeded on gelatin non-coated plates' using the protocol IV and ECM extraction was carried out using the same protocol.

### Quantification of Collagen and Protein Content

Collagen content was detected by picrosirius red staining followed by counter staining using haemotoxylin. Sirius red staining was quantified by dissolving the stain using 0.5 N Sodium Hydroxide (0.5 N NaOH) and the absorbance was measured at 560 nm using a Microplate Reader (Berthold technologies, USA). Protein content was determined by the Bicinchoninic Acid (BCA) assay using Pierce BCA Protein Assay Kit (Pierce, USA) with different concentration of bovine serum albumin (BSA) (Pierce, USA) as standards. The absorbance was read at 562 nm after incubation at 37°C for 20–30 min using Microplate Reader.

### Peptide/Protein Identifications by nLC-MS/MS Analysis

The proteins samples were reduced (DTT), alkylated (IAA) and then digested using trypsin, sequence grade, modified (Sigma Aldrich, USA) in 50 mM ammonium bicarbonate by incubating overnight at 37°C.The peptide samples were analyzed by nano-LC–MS^E^ (MS at elevated energy) using a nano ACQUITY UPLC System (Waters, Manchester, UK) coupled to a Quadrupole-Time of Flight (Q- TOF) mass spectrometer (SYNAPT-G2, Waters). 3 µL of sample was loaded into the reverse phase column with 0.1% formic acid in water as mobile phase A and 0.1% formic acid in acetonitrile as mobile phase B. The peptide separation was performed on a 75 µm×100 mm BEH C_18_ Column, with particle size of 1.7 µm and a gradient elution of 1–40% mobile phase B, for 55.5 min at 300 nL/min flow rate. The parameters used for Mass Spectrometry include: nano-ESI capillary voltage −3.2 KV; sample cone −35 V; extraction cone −4 V; transfer CE −4 V; trap gas flow −2 mL/min; IMS gas (N_2_) flow −90 mL/min; IMS T-Wave pulse height −40 V (during transmission) and the IMS T-Wave velocity −800 m/s.

### nLC-MS/MS Data Analysis

The LC- MS^E^ data was analyzed by using Protein Lynx Global SERVER v2.5.3 (PLGS; Waters, Manchester, UK). Data processing includes lock mass correction post acquisition. Noise reduction thresholds for low energy scan ion, high energy scan ion and peptide intensity combined across charge states and isotopes were fixed at 150, 50 and 500 counts respectively. *Mus musculus* (house mouse) database downloaded from NCBI was used for the database search. A peptide was required to have at least two assigned fragment and protein was required to have at least one assigned peptide and five assigned fragments for identification. Oxidation of methionine was selected as variable modification and cysteine carbamidomethylation was selected as a fixed modification. Trypsin was chosen as the enzyme used with specificity of 1 missed cleavage. Data sets were normalized using the ‘internal standard-normalization' function of PLGS and quantitative analyses was performed by comparing the normalized peak area/intensity of identified peptides between the samples. ADH was chosen as the internal standard for normalization during expression analysis. The proteins obtained were functionally clustered under different biological processes using the analysis software — DAVID (The Database for Annotation, Visualization and Integrated Discovery) [19].

### Cytocompatibility Studies

Cytocompatibility of cardiogel was assessed by seeding BMSCs on cardiogel and control plates (0.25% gelatin coated plates) at an initial density of ∼5×10^3^ cells per cm^2^. Cell viability was estimated using MTT (3-(4, 5- Dimethylthiazol-2-yl)-2,5-diphenyltetrazolium bromide) assay at different time points (24 h, 48 h and 72 h). Absorbance was measured at a wavelength of 570 nm with background subtraction at 650 nm using a Microplate Reader. The adhesion and migration of cells from cardiac explants were assessed by culturing the cardiac explants excised from neonatal mouse heart tissue on cardiogel and control plates at an average of ∼6 explants (∼1 mm^3^ each) per 10 cm^2^ for 15 days. The shedding of cells from the explant was monitored at different time points (Day 5, Day 10 and Day 15) using bright-field microscope (Nikon TiE, Melville, NY). All the experiments were performed in triplicates.

### Cardiomyogenic Differentiation of BMSCs on Cardiogel

BMSCs were seeded at an initial density of ∼ 5×10^3^ cells per cm^2^ and grown on cardiogel and control plates for 4 weeks without any chemical induction with replenishment of medium every 4 days. The morphological changes were photographed on Day 28 using bright-field microscope (Nikon TiE, Melville, NY). The experiment was repeated thrice and BMSCs cultured on 0.25% gelatin coated plates were used as control in this study. RNA and protein was isolated from the differentiated cells on Day 28, following which Real Time Polymerase Chain Reaction (RT-PCR) and Western Blotting analyses were carried out for cardiac-specific markers.

### Angiogenesis Studies on Cardiogel using EA.hy926 cells

Angiogenesis studies were carried out by seeding EA.hy926 cells at initial seeding density of ∼0.1×10^6^ cells per 20 cm^2^ on cardiogel and control plates without addition of any external growth factor for 2 days. The experiment was repeated thrice. EA.hy926 cells cultured on 0.25% gelatin coated plates and matrigel were used as negative and positive controls respectively. The morphological changes were observed and photographed on Day 2 using bright-field microscope (Nikon TiE, Melville, NY). RNA was isolated from the cells on Day 2, following which RT-PCR analysis was performed for angiogenesis-specific markers.

### Immunocytochemistry (ICC)

Immunocytochemistry was carried out as previously described [20]. The samples were fixed in 4% paraformaldehyde (PFA) and incubated in primary antibodies at 4°C overnight with subsequent incubation in FITC/Phycoerythrin conjugated secondary antibodies for 2 h at RT. The nuclei were counter stained with 5 µg/ml Hoechst 33342 (Sigma-Aldrich, USA) and visualized using Nikon Ti fluorescence microscope with NIS Elements Nikon Advanced Research (Ars) 3.0 imaging software. Primary antibodies against Collagen I (COLI; 1∶500; raised in rabbit; Abcam, USA), Collagen III (COLIII; 1∶500; raised in rabbit; Abcam, USA), Laminin (LAM; 1∶500; raised in rabbit; Abcam, USA), Fibronectin (FIB; 1∶500; raised in rabbit; Abcam, USA), Vimentin (VIM; 1∶500; raised in rabbit; Abcam, USA), DDR2 (1∶50; raised in rabbit; Santa Cruz, USA), THY1 (1∶150; raised in rabbit; Santa Cruz Biotechnology, USA), Periostin (POSTN; 1∶500; raised in rabbit; Abcam, USA), GATA4 (1∶150; raised in mouse; Santa Cruz Biotechnology, USA), SCA1 (1∶50; raised in rat; BD Biosciences, USA), CD44 (1∶50; raised in rat; BD Biosciences, USA), CD29 (1∶50; raised in rat; BD Biosciences, USA), CD106 (1∶50; raised in rat; BD Biosciences, USA) and CD34 (1∶50; raised in rat; BD Biosciences, USA) were used along with goat anti-rabbit, anti-mouse and anti-rat FITC/Phycoerythrin conjugated secondary antibodies (1∶250; Sigma Aldrich, USA).

### Real Time PCR (RT-PCR)

RNA was isolated using Trizol reagent (Sigma Aldrich, USA) according to the manufacturer's protocol. 1.5 µg of total RNA was converted to cDNA in a 20 µl reaction volume using MMLV-RT (Thermo scientific, USA) and oligo-dT primers (NEB, USA). RT- PCR was carried out as previously described [21] using Applied Biosystems SYBR Select Master mix on Applied Biosystems 7500 Real-Time PCR Systems (Life Technologies, USA). Briefly, a two-step cycling protocol was performed consisting of a single 10-min cycle at 95°C, followed by 40 cycles of 10 s at 95°C, and 30 s at 60°C. Melting curve analysis was performed after amplification to check for the presence of any spurious amplification or for the formation of primer dimers. Relative mRNA expression was determined by normalization to the expression of a housekeeping gene, Beta-actin (Primer list is provided in S1 Table).

### Western blotting

Western Blotting was performed as previously described [22]. Briefly, the protein samples were mixed with Laemmli sample buffer (1×) and boiled for 5 min. Proteins were subjected to 10% SDS-PAGE and electroblotted onto BioRad, 0.22 µM nitrocellulose membrane (BioRad Laboratories, USA), followed by blocking for 1 h at room temperature using Tris-buffered saline with 0.2% Tween 20 (TBS-T) containing 3% BSA. The membrane was incubated with primary antibodies at 4°C overnight and subsequently incubated with secondary antibodies conjugated with horseradish peroxidase (Sigma Aldrich, USA) for 2 h at RT and washed again with TBS-T. Antibody-reactive proteins were detected by enhanced chemiluminescence using Pierce ECL Plus western blotting detection reagents (Pierce, USA). Primary antibodies for GATA4 (1∶150; Santa Cruz Biotechnology, USA), Connexin43 (CX43; 1∶500; Abcam, USA), Alpha sarcomeric actin (ACTA1; 1∶500; Sigma Aldrich, USA) and Beta Actin (ACTB; 1∶1000; Sigma Aldrich, USA) were used along with goat anti-rabbit and anti-mouse horseradish peroxidase conjugated secondary antibodies (1∶10,000; Sigma Aldrich, USA).

### Statistical Analysis

All statistical analyses were performed using GraphPad Prism version 6 (GraphPad Software, Inc., USA). Differences among three or more groups were performed by analysis of variance (ANOVA) followed by Holm-Sidak test while Two tailed student's T-test (unpaired) was used for comparing two groups. P-values of ≤0.05 indicate significant differences. Error bars are given for data representing average and standard deviation in case of two or more independent experiments. Experimental data are shown for experiments performed in triplicates.

## Results

### Optimization of ECM extraction

**Figure 1:**
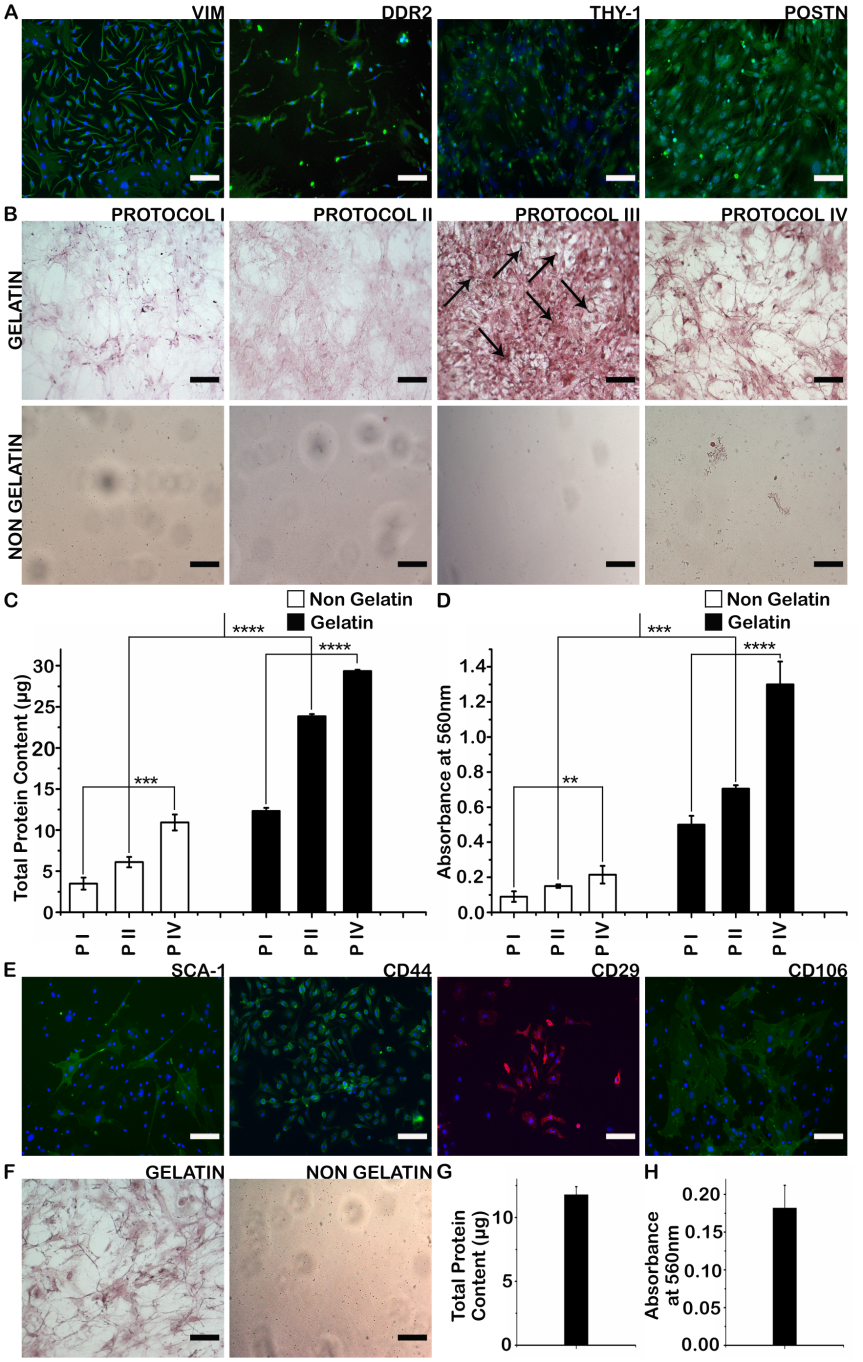
Optimization of cardiogel and mesogel extraction. (A): Immunocytochemistry (ICC) of cardiac fibroblasts for a panel of surface markers: Vimentin, DDR2, Thy-1 and Periostin (B) Picrosirius Red Staining and Hematoxylin counter-staining of cardiogel obtained by the four different decellularization protocols on gelatin coated and non-coated plates. Arrows indicate hemotoxylin-stained nuclei (C) Comparison of protein yield from cardiogel obtained by the different decellularization protocols (D) Quantification of Picrosirius Red Staining from cardiogel obtained by the different decellularization protocols (E) Immunocytochemistry (ICC) of BMSCs for a panel of surface markers: Sca-1, CD44, CD29 and CD106 (F) Picrosirius Red Staining of mesogel obtained by the optimized protocol (G) Protein yield from mesogel obtained by the optimized protocol (H) Quantification of Picrosirius Red Staining from mesogel obtained by the optimized protocol. Scale bar  = 100 µm; All results are expressed as average and standard deviation in case of three independent experiments n = 3 (mean ± S.D); **p<0.01, ***p<0.001, ****p<0.0001; Abbreviations: VIM, Vimentin; POSTN, Periostin; PI, Protocol I; PII, Protocol II; PIV, Protocol IV.

Cardiac fibroblasts were positive for a panel of surface markers which included characteristic proteins such as DDR2 and Periostin (Fig. 1A) and negative for cardiomyocyte-specific marker GATA4 (S1A Figure). Decellularization of cardiac fibroblast cultures was carried out using four different protocols on gelatin coated and non-coated plates to generate cardiogel. Picrosirius red staining for collagen content in cardiogel revealed the presence of thin collagen fibers on gelatin coated plates, whereas collagen fibres appeared like granules scattered throughout the surface of the non-coated plates (Fig. 1B). Protein and collagen yield from cardiogel was higher on gelatin coated plates than non-coated plates (Fig. 1C and D), suggesting that gelatin coating preserves the structure of the decellularized matrix resulting in a higher yield of ECM. To examine the presence of any nuclear contamination following decellularization with the four protocols, hematoxylin staining of cardiogel was performed. Since, hematoxylin stained nuclei were present in cardiogel from protocol III, indicating incomplete decellularization (Fig. 1B), protocol III was excluded from the subsequent studies. Protein quantification showed that protocol IV yielded highest protein among all protocols tested (Fig. 1C). Quantification of collagen content using picrosirius red staining also yielded similar results (Fig. 1D). Therefore, protocol IV was chosen for all further studies.

BMSCs were positive for a panel of surface markers, which included characteristic proteins such as Sca-1 and CD44 (Fig. 1E) and negative for endothelial-specific marker CD34 (S1B Figure). Mesogel was then isolated and extracted from BMSC cultures using protocol IV, generating similar results as obtained with cardiogel (Fig. 1F, G and H), establishing the robustness of the developed protocol.

### Preliminary characterization of cardiogel and mesogel

**Figure 2:**
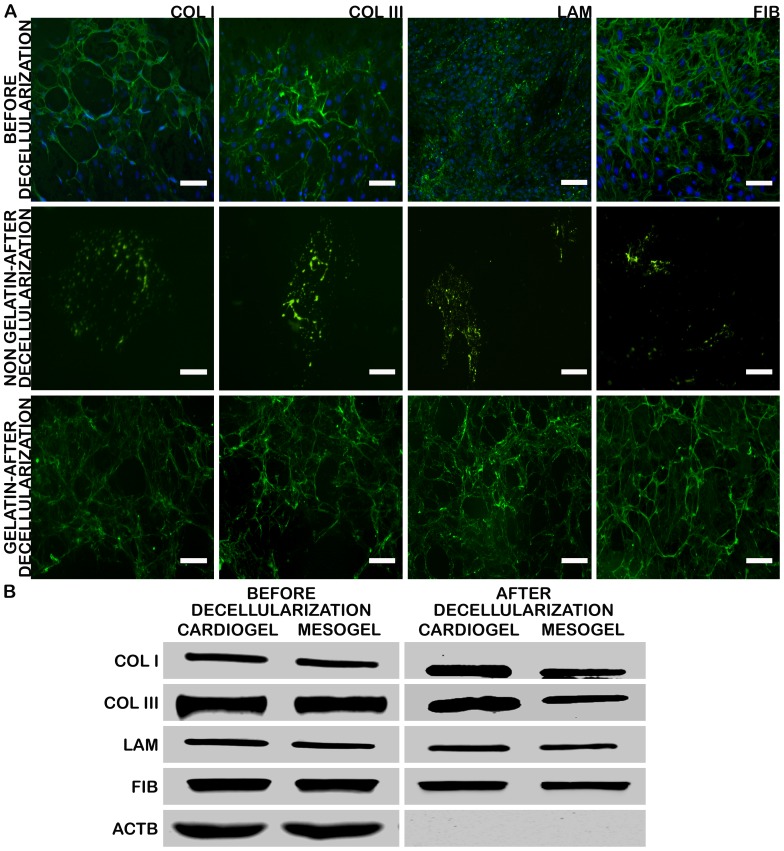
Preliminary characterization of cardiogel and mesogel. (A) Immunocytochemistry (ICC) of cardiogel for a panel of ECM structural proteins before and after decellularization on gelatin coated and non-coated plates, counter-stained with Hoechst staining for nucleus (B) Western Blotting of cardiogel and mesogel for a panel of ECM structural proteins before and after decellularization with ACTB as internal control. Scale bar  = 100 µm; Abbreviations: COLI, Collagen Type I; COLIII, Collagen Type III; LAM, Laminin; FIB, Fibronectin; ACTB, Beta Actin.

Preliminary analysis of cardiogel using Immunocytochemistry identified that cardiogel was made up of several structural proteins such as Collagen Type I, Collagen Type III, Laminin and Fibronectin (Fig. 2A). Western blotting analysis, with Beta Actin (B-Actin) as the internal control, revealed that the amount of Collagen I, Collagen III, Laminin and Fibronectin was relatively high in cardiogel in comparison to mesogel (Fig. 2B). Further, nuclear staining with Hoechst and western blotting for B-Actin, a cytoskeletal protein, were negative in cardiogel and mesogel after decellularization (Fig. 2A and Fig. 2B). These results indicated that the optimized protocol yielded the highest amount of ECM with complete removal of all intracellular components resulting in efficient decellularization and no prevalent cellular contaminations.

### Proteins differentially identified in cardiogel and mesogel

**Figure 3:**
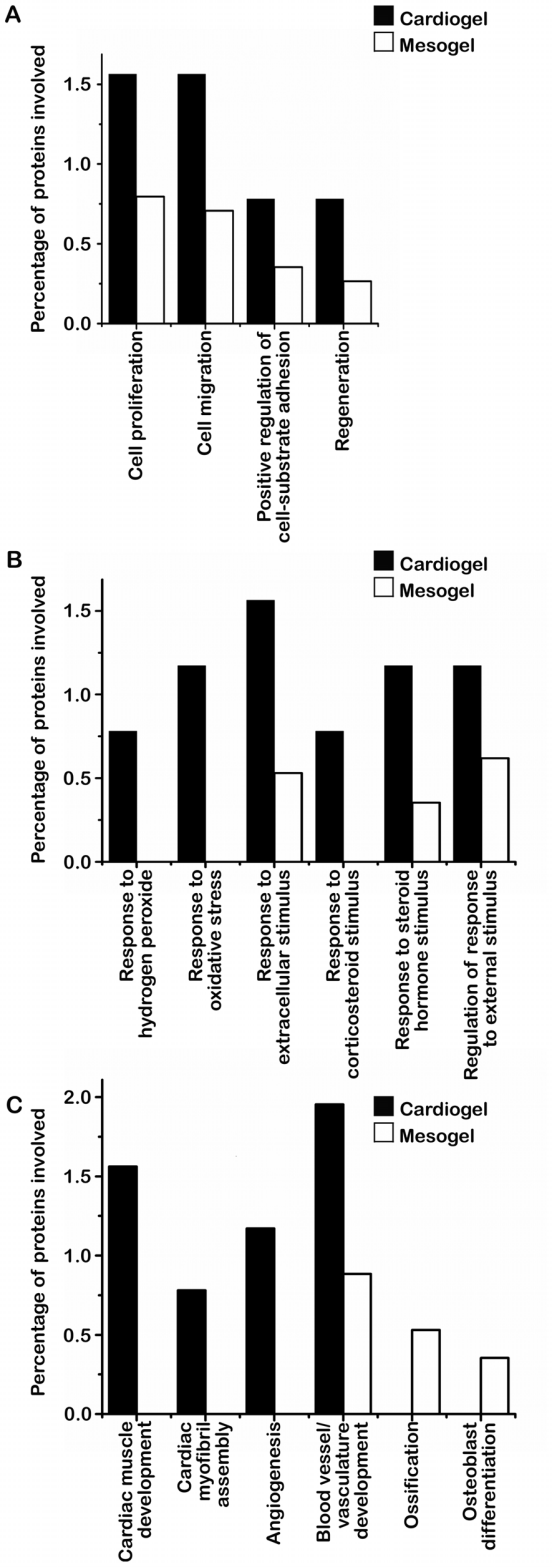
Functional clustering of proteins identified in cardiogel and mesogel by nLC-MS/MS analysis. (A) Functional clustering of proteins involved in cellular functions (B) Functional clustering of proteins involved in response to stimulus (C) Functional clustering of proteins involved in differentiation and regeneration processes; Functional annotation clustering was carried out for the 265 unique proteins identified by nLC-MS/MS using DAVID software; Proteins (%) represents the percentage fraction of proteins within each biological process with reference to a total number of 265 proteins identified by nLC-MS/MS analysis.

Comparative proteomic analysis of cardiogel and mesogel using a label-free spectral counting approach in three independent nLC-MS/MS runs identified 265 unique proteins. These proteins were normalized using Protein Lynx Global Server (PLGS 2.2.5) software with a minimum PLGS Score of 200 and the protein false positive rate of 4% set as searching and validation criteria. Such stringent criteria provided greater confidence in the identified proteins. Only the proteins, which appeared in all three independent repeats, were considered for further analysis. Functional clustering using DAVID software identified that the percentage of proteins involved in biological processes such as cell proliferation, cell migration, response to extracellular stimuli and blood vessel/vasculature development were higher in cardiogel compared to mesogel (Fig.3A, B and C, S2 Table). Further, biological processes such as response to oxidative stress/hydrogen peroxide, cardiac muscle tissue development and angiogenesis were specific to cardiogel while muscle development and osteogenesis were specific to mesogel (Fig.3B and C, S2 Table). Among proteins present in these biological processes, 17 significant proteins were differentially identified between cardiogel and mesogel (Table 1 and Table 2), including proteins such as Thymosin beta 4, Alpha 2 macroglobulin, Osteoprotegerin and Annexin A2. The results of these analyses may explain the different biological properties of cardiogel such as increased cell proliferation, adhesion, cardiomyogenic differentiation, protection against oxidative stress, angiogenesis as well as inhibition of osteogenesis.

**Table 1:** Proteins functioning in significant biological processes in cardiogel.

Accession Number	Proteins	PLGS Score	Functions
P20065	Thymosin beta 4 (Tmsb4x)	2212.47	Protection against oxidative stress, cardiac apoptosis and fibrosis; Cardiomyogenic differentiation of ESCs and MSCs; Angiogenesis; Myocardial and endothelial cell migration, survival and repair; Retention of transplanted MSCs
Q61838	Alpha 2 macroglobulin (A2m)	300.29	Differentiation of ESCs into endothelial and early cardiac muscle; Hypertrophy and contractile response of ventricular cardiomyocytes
P19246	Neurofilament heavy polypeptide (Nefh)	211.83	Structural components of the cytoskeleton in specialized myocytes
P08553	Neurofilament medium polypeptide (Nefm)	280.77	Structural components of the cytoskeleton in specialized myocytes
Q61207	Sulfated glycoprotein 1/Prosaposin (Psap)	372.44	Neurotrophic and Anti-apoptotic factor present in heart
P28776	Indoleamine 2, 3 dioxygenase 1 (Ido1)	300.02	Cardiac allograft survival and immunological tolerance

**Table 2:** Proteins functioning in significant biological processes in mesogel.

Accession Number	Proteins	PLGS Score	Functions
P19324	Serpin H1 (Hsp47/Serpinh1)	549.1	Cartilage and endochondral bone formation; Endochondral ossification.
O08712	Tumor necrosis factor receptor superfamily member 11B/Osteoprotegerin (Tnfrsf11b/OPG)	916.56	Osteogenesis of mesenchymal stem cells
P11499	Heat shock protein 90 beta (Hsp90ab1)	710.13	Multipotent mesenchymal precursor cell surface marker
P46660	Alpha internexin (Ina)	1427.3	Neuronal intermediate filament protein
P11152	Lipoprotein lipase (Lpl)	318.08	Early marker of adipocyte differentiation
P13020	Gelsolin (Gsn)	301.93	Assembly and disassembly of actin filaments; Platelet activation
P07356	Annexin A2 (Anxa2)	782.94	Osteoblastic mineralization
Q61879	Myosin 10 (Myh10)	518.08	Muscle contraction; Megakaryocyte polyploidization and differentiation
O08638	Myosin 11 (Myh11)	380.26	Muscle cell maturation; Muscle contraction
P16627	Heat shock 70 kDa protein 1 like (Hspa1l)	797.55	Protein stabilization against aggregation; Protein folding
Q60930	Voltage dependent anion selective channel protein 2 (Vdac2)	444.13	Regulation of mitochondrial apoptosis

### Cytocompatibility analysis of cardiogel

**Figure 4:**
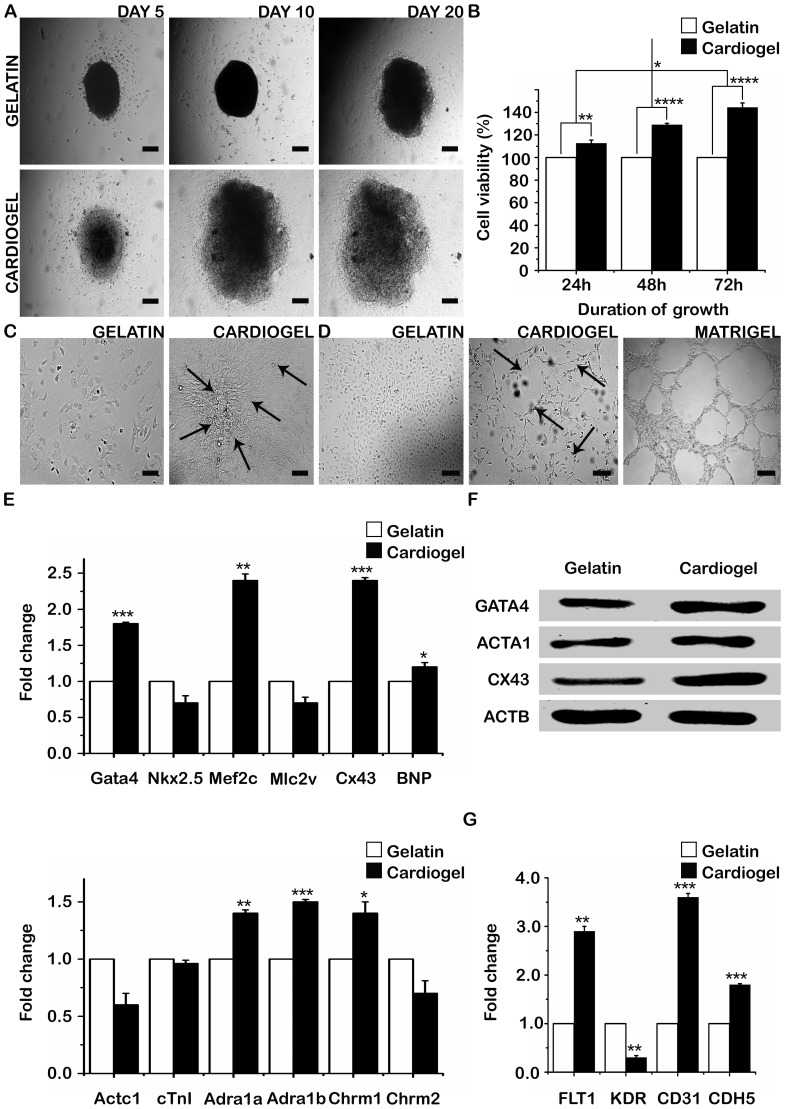
Analysis of regenerative potential of cardiogel. (A) Cardiac explants cultured on cardiogel and gelatin coated controls (B) Cytocompatibility studies on cardiogel by MTT Assay (C) Cardiomyogenic differentiation of BMSCs on cardiogel and gelatin coated controls. Arrows indicate multi-nucleation and three-dimensional myotubule-like formation (D) Angiogenesis studies on cardiogel and gelatin coated controls by *in vitro* tube formation assay. Arrows indicate formation of capillary-like structures and polygon structures (E) Quantitative RT-PCR analysis for a panel of cardiomyogenic differentiation markers, normalized using Actb as internal control. Results were expressed as ratio of fold change in mRNA expression in BMSCs cultured on cardiogel compared to gelatin coated controls (F) Western Blotting for cardiac markers in BMSCs cultured on cardiogel compared to gelatin coated controls. ACTB was used as internal control (G) Quantitative analysis for a panel of angiogenic markers, normalized using ACTB as internal control. Results were expressed as ratio of fold change in mRNA expression in EA.hy926 cells cultured on cardiogel compared to gelatin coated controls; Scale bar  = 100 µm; All results are expressed as average and standard deviation in case of three independent experiments (n = 3; mean ± S.D); *p<0.05, **p<0.01, ***p<0.001; Abbreviations: Gata4/GATA4, GATA binding protein 4; Nkx2.5, NK2 homeobox 5; Mef2c, Myocyte-specific enhancer factor 2C; Mlc2v, Myosin light chain 2v; Cx43/CX43, Connexin 43; BNP, Brain natriuretic peptide; Actc1, Alpha cardiac muscle actin 1; cTnI, Cardiac Troponin I; Adra1a/1b, Adrenergic receptor, alpha 1a/1b; Chrm1/2, Cholinergic receptor, muscarinic 1/2; Actb/ACTB, Beta Actin; ACTA1, Alpha sacromeric actin; FLT1, FMS-related tyrosine kinase 1; KDR, Kinase insert domain receptor; CDH5, Cadherin 5.

Cardiac explants derived from neonatal mouse hearts adhered faster and the cells began to shed and migrate at a faster rate on cardiogel in comparison to gelatin coated controls (Fig. 4A). Comparison of cell viability/proliferation by MTT Assay demonstrated that BMSCs cultured on cardiogel were viable and showed a significant increase in the proliferation rate as compared to gelatin coated controls. In addition, there was a gradual increase in the proliferation rate with increase in the duration of culture (24, 48 and 72 h) (Fig. 4B).

### Regenerative potential of stem cells and endothelial cells cultured on cardiogel

To evaluate the regenerative capabilities, the cardiomyogenic differentiation and angiogenic potential of stem cells and endothelial cells on cardiogel were examined. Both processes were carried out without addition of any external growth factors or chemical inducers. When BMSCs were cultured on cardiogel, approximately 15–20% of the cells developed three-dimensional myotubule-like multinucleated structures by the end of 4^th^ week (Fig. 4C). Initial stages of angiogenesis such as EC sprouting, capillary-like structure and polygon structure formation were observed when EA.hy926 cells were cultured on cardiogel by Day 2 (Fig. 4D). RT-PCR analysis and Western Blotting for cardiac and angiogenic markers further validated these observations. RT-PCR analysis of BMSCs showed a significant increase in the expression of cardiac markers such as Gata4, Mef2c, Cx43, BNP, Adra1a, Adra1b and Chrm1 (Fig. 4E) and Western Blotting showed an increase in the expression of GATA4, ACTA1 and CX43 in BMSCs cultured on cardiogel in comparison to those cultured on gelatin coated controls (Fig. 4F). RT-PCR analysis of EA.hy926 cells showed an increased expression of FLT1 (VEGFR1), CD31 and CDH5 (VE-cadherin/CD144) in cells cultured on cardiogel compared to those cultured on gelatin coated controls (Fig. 4G).

## Discussion

Previous studies have described the role of the extracellular matrix in stem cell differentiation and myocardial tissue organization. The cardiac ECM comprises of several proteins and polysaccharides, which play a crucial role in development and remodeling of heart [23], [24]. We generated a natural heterogeneous ECM scaffold, cardiogel, from cultured cardiac fibroblasts. This scaffold has been shown to promote proliferation, adhesion, cardiomyogenic differentiation of BMSCs and provide protection against oxidative stress [12]–[17], but the composition of this complex matrix has not yet been completely characterized. In this study, comparative proteomic analysis of cardiogel was performed to obtain a detailed characterization of this nano-matrix. However, literature describing the extraction of an ECM from *in vitro* cultured cells and its proteomic analysis is limited [25]. An accurate and reliable proteomic analysis of any ECM requires a substantial amount of protein, absence of any interfering substances such as detergents and intracellular contaminations, and complete solubilization of the fibrous proteins in the matrix [21]. These exacting requirements necessitate an optimized protocol for the efficient decellularization, extraction and solubilization of an ECM. Decellularization protocols using several non-enzymatic agents have been widely used to generate ECM scaffolds; but, protocols using cation chelating agents such as EDTA do not remove the cells completely while ECM extracted with methods using NH_4_OH and TX100 have been reported to contain intracellular impurities [25], [26]. Therefore, in the present study, four different decellularization protocols with various combinations and concentrations of the above reagents were used for generation of cardiogel. Protocol IV resulted in complete decellularization with maximum yield of ECM and was chosen for further studies.

Previous studies have used gelatin as a substrate for cardiogel isolation [15], [16]. To understand the effect of gelatin during decellularization of cardiac fibroblasts, four different protocols were carried out on cells cultured on gelatin coated and non-coated plates. It was observed that gelatin coating not only preserved the structure of the matrix but also yielded higher amount of protein and collagen compared to gelatin non-coated plates. These effects can be attributed to presence of gelatin, which can enhance the binding of ECM to the plates preventing the matrix from being dislodged during decellularization. However, LC-MS analysis of pure gelatin revealed the presence of several matricellular proteins [27] and hence the presence of gelatin in decellularized matrix during proteomic analysis of cardiogel might lead to unreliable results. Therefore, cardiogel from non-coated plates were used for proteomic analysis, while cardiogel from gelatin coated plates were used in studies evaluating its biological properties such as cytocompatibility and regenerative potential.

Fibrous proteins such as collagen and fibronectin are cross-linked in ECM and usually insoluble during protein extraction [21]. A two step ECM solubilization process, which included treatment with 5% acetic acid followed by buffer containing SDS and DTT, was used to facilitate the solubilization of these matrix proteins. However, the presence of detergents such as SDS can interfere during proteomic analysis and therefore such detergents were removed using acetone precipitation. Finally, surfactant-assisted solubilization of the protein pellet using 0.1% Rapigest SF surfactant was carried out to ensure complete solubilization of cardiogel. The same extraction protocol was followed for extraction of mesogel, which was used as control ECM for cardiogel in proteomic analysis.

Preliminary characterization showed that the levels of Collagen I and III, Laminin and Fibronectin were relatively high in cardiogel as compared to mesogel. This difference in the proportion of ECM structural proteins may contribute to the observed biological properties in cardiogel. An in-depth comparison of cardiogel and mesogel using label-free nLC-MS/MS followed by functional clustering using DAVID, revealed that proteins involved in biological processes such as cardiac muscle development, angiogenesis and response to oxidative stress/hydrogen peroxide were unique to cardiogel while those involved in osteogenesis and muscle development were specific to mesogel. In addition, the percentage of proteins involved in cell proliferation, cell migration, positive regulation of cell-substrate adhesion, regeneration, response to extracellular/steroid hormone/external stimuli and blood vessel/vasculature development were higher in cardiogel compared to mesogel. These results corroborated with the properties of cardiogel and mesogel [12]–[17], [28]–[30].

Tmsb4x and A2m have been reported to enhance the differentiation of Embryonic Stem Cells (ESCs) into functional cardiomyocytes [31], [32]. Cardiogel has also been shown to promote early differentiation and maturation of ESC-derived cardiomyocytes [14], [33]. Tmsb4x and A2m have also been shown to stimulate angiogenesis by promoting endothelial cell migration, survival and differentiation [32], [34], [35]. Tmsb4x and A2m were identified in the proteomic analysis, suggesting a role for these proteins in the differentiation and angiogenic potential of cardiogel.

Cardiogel has been shown to provide a substrate environment for growth and maturation of cardiomyocytes by influencing their spontaneous contractile activity and phenotype morphological differentiation [12], [13], [36]. This feature could be explained by the presence of proteins such A2m, which was known to improve contractile response of ventricular cardiomyocytes [37] and Nefh and Nefm that were observed to be structural components of the cytoskeleton in specialized myocytes [38]. Proteins such as Tmsb4x and Psap are known to protect cardiomyocytes against oxidative stress and apoptosis [31], [39]–[41]. Similarly, cardiogel also possessed protective properties against hypoxic conditions [13]. Additionally, Tmsb4x and Ido1 were found to improve the retention and survival of cardiac graft after transplantation following MI [42], [43], which was also observed in cardiogel [44]. Tmsb4x has been known to inhibit osteogenic differentiation but reciprocally facilitate adipogenic differentiation [45]. Similarly cardiogel has been observed to promote adipogenesis of BMSCs while osteogenic differentiation was found to be insignificant [14]. Tmsb4x was also known to aid in murine cardiac development, which was also observed in cardiogel [46], [24].

Among the proteins identified in mesogel, Hsp47, Tnfrsf11b, Anxa2, Myh10 and Myh11 were observed to promote differentiation of MSCs into osteogenic, chondrogenic or myogenic lineages [47], [48] while Ina and Lpl were found to be markers of differentiation into neuronal and adipogenic lineages [49]–[52]. The absence of these proteins in cardiogel could have contributed to the enhanced cardiomyogenic differentiation and decreased osteogenesis that was observed in cardiogel [14]. Few proteins like Gsn, Vdac2 and Hspa1l were known to be involved in general cellular component organization such as assembly and disassembly of actin filaments, regulation of mitochondrial apoptosis, protein stabilization against aggregation and protein folding respectively [53]–[55].

Biomaterials to be used as scaffolds for cardiac tissue construct, should be biocompatible, and should exhibit functional and morphological properties of native heart [56], [57]. Cytocompatibility studies proved that cardiogel increased cell proliferation, adhesion and migration. These effects could be attributed to the synergistic effect of proteins such as Tmsb4x and Psap, which were known to improve cell survival and migration and also prevent apoptosis of cardiomyocytes under stress [35], [38], [39]. Cardiogel also promoted cardiomyogenic differentiation of BMSCs. Previous studies have reported that 5-azaC treatment induces BMSCs to elongate and develop into myotubule-like multinucleated structures [33]. However, similar morphological changes were observed in BMSCs cultured on cardiogel, even in the absence of any chemical induction. RT-PCR analysis showed an increased expression of cardiac transcription factors such as Gata4 and Mef2c, the major cardiac gap junction protein Cx43, a cardiac hormone BNP, the adrenergic receptor Adra1a and Adra1b and the muscarinic acetylcholine receptor, Chrm1 while Western Blotting showed an increase in the expression of GATA4, ACTA1 and CX43 in BMSCs cultured on cardiogel compared to those cultured on gelatin coated controls. The cardiomyogenic differentiation of BMSCs on cardiogel could be contributed to the presence of proteins such as Tmsb4x, A2m, laminin and fibronectin, which are known to enhance cardiomyogenic differentiation of stem cells [31], [32], [58], [59].

Although cardiogel does not induce complete tubulogenesis, it was found to support initial stages of angiogenesis such as Endothelial Cell (EC) sprouting, formation of capillary-like structures and polygon structures in the absence of any external angiogenic factors. Previous studies have suggested that, since angiogenesis is a highly complex and multi-factorial process, its measurement should not be limited to only investigating the formation of tubular structures but include cell proliferation, migration, EC sprouting and capillary-like structures formation [60]. The above features were also observed in cells cultured on cardiogel. In addition, RT-PCR analysis showed that there was an increased expression of angiogenic markers such as FLT1, CD31 and CDH5 in EA.hy926 cells cultured on cardiogel in comparison to gelatin coated controls. The angiogenic potential of cardiogel could be attributed to the synergistic effect of various proteins such as collagens I and III, laminin, fibronectin, Tmsb4x and A2m which are known to promote endothelial cell proliferation, migration and neo-vasculogenesis [32], [34], [35], [61]–[68].

## Conclusion

We have developed a reproducible protocol for the efficient decellularization, extraction and solubilization of ECM from cultures of cardiac fibroblasts and BMSCs. The protocol allows the generation of cardiogel as a nanomatrix following decellularization, and also allows the subsequent extraction and solubilization of the matrix for possible use in conjunction with other biomaterials. The present study has identified for the first time, several extracellular matrix proteins as well as matricellular proteins, which can act as potential biochemical stimuli which contribute to the biological properties of cardiogel including cardiomyocyte growth and maturation, cardiomyogenic differentiation, protection against oxidative stress and angiogenesis. Cardiogel promotes cell proliferation, adhesion and migration while aiding cardiomyogenic differentiation and angiogenesis. In conclusion, cardiogel possesses key biological properties that are essential for three-dimensional cardiac tissue constructs. Such constructs based on cardiogel will have potential for application in cardiac regeneration and repair using stem cells.

## Supporting Information

S1 Figure **Immunocytochemistry (ICC) for negative markers.** (A): Immunocytochemistry (ICC) of cardiac fibroblasts for a negative (control) marker, GATA4 (B) Immunocytochemistry (ICC) of BMSCs for a negative (control) marker, CD34; Scale bar  = 100 µm. Abbreviations: GATA4, GATA binding protein 4.
(TIF)

S1 Table **List of primers.**
(DOC)

S2 Table **Functional enrichment of proteins identified by nLC-MS/MS.**
(DOC)
