# Supplementary figures and images for: Cardiogel: A Nano-Matrix Scaffold with Potential Application in Cardiac Regeneration Using Mesenchymal Stem Cells
Source: PLoS One. 2014 Dec 18;9(12):e114697. doi: 10.1371/journal.pone.0114697 (PMC4270637; doi:10.1371/journal.pone.0114697)

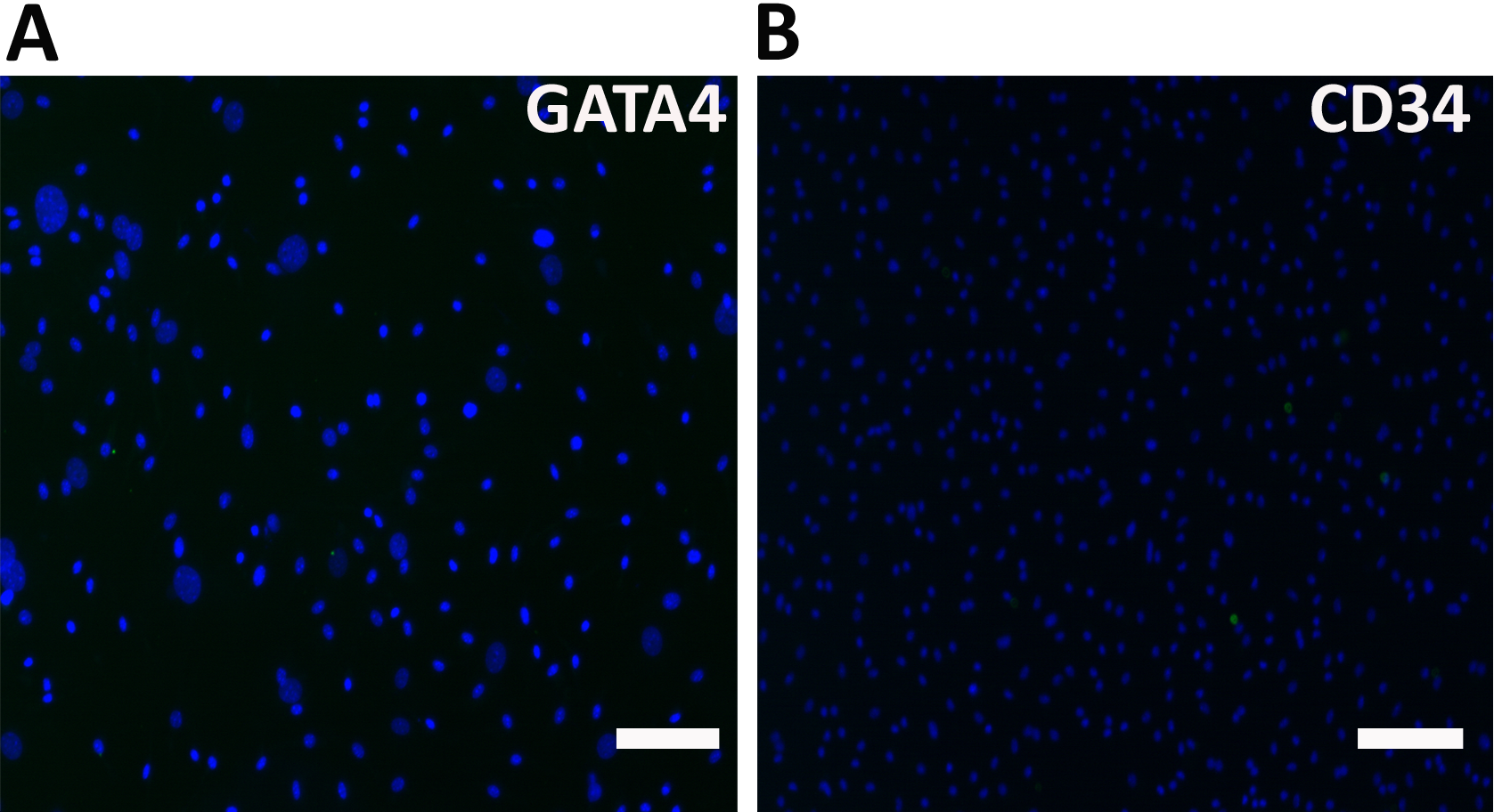

Supplement: S1 Figure — Immunocytochemistry (ICC) for negative markers. (A): Immunocytochemistry (ICC) of cardiac fibroblasts for a negative (control) marker, GATA4 (B) Immunocytochemistry (ICC) of BMSCs for a negative (control) marker, CD34; Scale bar = 100 µm. Abbreviations: GATA4, GATA binding protein 4. (TIF) [file pone.0114697.s001.tif]
