# Supplementary material for: Cardiogel: A Nano-Matrix Scaffold with Potential Application in Cardiac Regeneration Using Mesenchymal Stem Cells
Source: PLoS One. 2014 Dec 18;9(12):e114697. doi: 10.1371/journal.pone.0114697 (PMC4270637; doi:10.1371/journal.pone.0114697)
Supplement: S1 Table — List of primers. (DOC) [file pone.0114697.s002.doc]

**Table S1. List of primers**

| **Primer**  **(Product size in base pairs (bp))** | | **Sequence**  **(Forward Primer (FP); Reverse Primer (RP))** |
| --- | --- | --- |
| **Species: *Mus musculus*** | | |
| Actb (197 bp) | | FP 5’- CTT CTT TGC AGC TCC TTC GTT -3’  RP 5’- TTC TGA CCC ATT CCC ACC A -3’ |
| Actc1 (97 bp) | | FP 5'- CTG AGA TGT CTC TCT CTT AG -3'  RP 5'- CAA TGA CTG ATG AGA GAT GG -3' |
| Adra1a (158 bp) | | FP 5’- CGT GAC TGG TAA GCA AGC AA-3’  RP 5’- CAA ACC ATC ACC TCC AGC TT-3’ |
| Adra1b (191 bp) | | FP 5’- CGC CCA CCA ACT ACT TCA TT-3’  RP 5’- AAT GGA GAT GGC ACA TAG GC-3’ |
| BNP (210 bp) | | FP 5’- ATG GAT CTC CTG AAG GTG CT -3’  RP 5’- AAG AGG GCA GAT CTA TCG GA -3’ |
| Chrm1 (198 bp) | | FP 5’- AGA AGA GGC TGC CAC AGGTA-3’  RP 5’- CAG ACC CCA CCT GGA CTT TA-3’ |
| Chrm2 (192 bp) | | FP 5’- TAA AGT CAA CCG CCA CCT TC-3’  RP 5’- ATA ACG GAG GCA TTG CTG AC-3’ |
| cTnI (126 bp) | | FP 5'- CGG GCG TTG GAA ATA GAT G -3'  RP 5'- TCC CAC TAT CCA AAC AGG AG -3' |
| Cx43 (210 bp) | | FP 5’- TCC CAC GGA GAA AAC CAT C -3’  RP 5’- GAG CAG CCA TTG AAG TAA GC -3’ |
| Gata4 (257 bp) | | FP 5'- CTG TCA TCT CAC TAT GGG CA -3'  RP 5'- CAA GTC CGA GCA GGA ATT TG -3' |
| Mef2c (125bp) | | FP 5'- GTG CCA ACA AAA GCA TTG AA -3'  RP 5'- AGC TCA GTT CCC AAA TCC CT -3' |
| Mlc2v (261 bp) | | FP 5'- TGT TCC TCA CGA TGT TTG GG -3'  RP 5'- CTC AGT CCT TCT CTT CTC CG -3' |
| Nkx2.5 (215 bp) | | FP 5'- AGT GGA GCT GGA CAA AG -3'  RP 5'- TAG CGA CGG TTC TGG AAC -3' |
| **Species: *Homo sapiens*** | | |
| ACTB (285 bp) | FP 5’- AGC GAG CAT CCC CCA AAG TT -3’  RP 5’- GGGCACGAAGGCTCATCATT- 3’ | |
| CD31 (151 bp) | FP 5’- TCC CCT AAG AAT TGC TGC CA -3’  RP 5’- TTC TTC CCA ACA CGC CAA TG -3' | |
| CDH5 (224 bp) | FP 5’- TAC CAG GAC GCT TTC ACC AT -3’  RP 5’- AAA GGC TGC TGG AAA ATG GG -3’ | |
| FLT1 (211 bp) | FP 5’- GTC GTG TAA GGA GTG GAC CA -3’  RP 5’- GCA GAT TTC TCA GTC GCA GG -3’ | |
| KDR (170 bp) | FP 5’- TTA CTT GCA GGG GAC AGA GG -3’  RP 5’- TTC CCG GTA GAA GCA CTT GT -3’ | |
