# Supplementary material for: Cardiogel: A Nano-Matrix Scaffold with Potential Application in Cardiac Regeneration Using Mesenchymal Stem Cells
Source: PLoS One. 2014 Dec 18;9(12):e114697. doi: 10.1371/journal.pone.0114697 (PMC4270637; doi:10.1371/journal.pone.0114697)
Supplement: S2 Table — Functional enrichment of proteins identified by nLC-MS/MS. (DOC) [file pone.0114697.s003.doc]

**Table S2. Functional enrichment of proteins identified by n**LC-MS/MS

| **Biological process** | **Proteins (%)** | |
| --- | --- | --- |
|  | **Cardiogel** | **Mesogel** |
| Cytoskeleton Organization | 17.58 | 19.45 |
| Metabolism | 2.34 | 11.94 |
| Organization of macromolecules and sub-cellular structures | 2.34 | 29.89 |
| Embryonic development | 19.53 | 8.58 |
| Cellular functions (Cell proliferation, adhesion, migration, regeneration, etc) | 11.72 | 6.10 |
| Regulation of cellular functions (Regulation of cell proliferation, adhesion, etc) | 30.86 | 10.70 |
| Response to external stimuli (Response to oxidative stress, corticosteroid/hormone stimulus, wounding, etc) | 16.41 | 3.45 |
| Extracellular matrix organization | 8.20 | 1.68 |
| Cardiac muscle development | 2.34 | -- |
| Angiogenesis and/or Blood vessel development | 5.08 | 2.30 |
| Muscle development | -- | 6.28 |
| Osteogenesis | -- | 1.68 |
| Blood coagulation | -- | 1.42 |
